# ILDR1 promotes influenza A virus replication through binding to PLSCR1

**DOI:** 10.1038/s41598-022-12598-3

**Published:** 2022-05-20

**Authors:** Yueyue Liu, Shuqian Lin, Yunhui Xie, Lu Zhao, Haibo Du, Shifa Yang, Bin Yin, Guiming Li, Zengcheng Zhao, Zhongli Huang, Zhigang Xu, Jiaqiang Wu

**Affiliations:** 1 Institute of Poultry Science, Shandong Academy of Agricultural Science, Shandong Provincial Animal and Poultry Green Health Products Creation Engineering Laboratory, Jinan, 250100 Shandong China; 2 Shandong Provincial Key Laboratory of Animal Cells and Developmental Biology, School of Life Sciences, Shandong University, Qingdao, 266237 Shandong China; 3 Shandong Provincial Collaborative Innovation Center of Cell Biology, Shandong Normal University, Jinan, 250014 Shandong China; 4 Key Laboratory of Animal Resistant Biology of Shandong, College of Life Sciences, Shandong Normal University, Jinan, 250014 Shandong China

**Keywords:** Infectious diseases, Molecular biology

## Abstract

As a natural antiviral regulator, phospholipid scramblase 1 (PLSCR1) has been shown to inhibit influenza virus replication in infected cells through interacting with NP of influenza A virus (IAV). But its antiviral function as well as the underlying regulatory mechanism has not been examined in vivo. In the present work, we show that PLSCR1 expression is decreased in H1N1 SIV-infected mice, and *Plscr1*^−/−^ mice are more susceptible to H1N1 SIV infection. By performing yeast two-hybrid screening, we identified immunoglobulin-like domain-containing receptor 1 (ILDR1) as a novel PLSCR1-binding partner. ILDR1 is highly expressed in the lungs, and its expression level is increased after virus infection. Interestingly, ILDR1 could not directly interact with virus NP protein, but could combine with PLSCR1 competitively. Our data indicates that there is a previously unidentified PLSCR1-ILDR1-NP regulatory pathway playing a vital role in limiting IAV infection, which provides novel insights into IAV-host interactions.

## Introduction

Influenza A viru (IAV) is an enveloped RNA virus of the Orthomyxovirus genus which causes serious deaths and economic losses^1^. The swine-derived H1N1 influenza that broke out in 2009 caused severe flu-like symptoms with a variety of complications in humans, and was a substantial global health concern^2^. Swine have receptors for both human influenza (SAα-2,6-Gal) and avian influenza (SAα-2,3-Gal), hence are referred to as "mixers" of influenza viruses^3^. They play an important role in the recombination of influenza viruses. It is therefore important to understand the pathogenic mechanisms and how these may be used to prevent and control of swine influenza virus (SIV) infection.

The negative-sense, single-stranded genome of IAV comprises eight segments of viral RNA, which are separately encapsidated into ribonucleoprotein particles (vRNPs). The virial RNA (vRNA) is bound to RNA-dependent RNA polymerase complex (RdRp) and encapsidated by the nucleoprotein (NP), forming the so-called vRNP complex^4^. The vRNP is the key to IAV life cycle and important for viral pathogenicity and host range determinants^5^. NP is essential for the translocation of vRNP into the nucleus of host cells and could be used as a target to block the replication of influenza virus specifically^6,7^. A series of host proteins have been reported to regulate nuclear entry through interacting with NP, such as α-actinin-4^8^, CRM1^9^, UAP56^10^, Hsp40^11^ and MOV10^12^, hence played important roles in promoting or inhibiting virus replication. So, identification of new NP-binding host proteins will help to provide new targets for prevention and/or control of influenza viruses.

Phospholipid scramblase 1 (PLSCR1) was first identified as a calcium-binding type II membrane protein that could be induced by interferons and growth factors^13^. It is involved in multiple biological processes, such as gene transcription regulation, cell proliferation, differentiation and apoptosis^14–17^. Recently, the antiviral activity of PLSCR1 received extensive attentions. It has been reported that PLSCR1 could inhibit HBV infection and replication through mediating ubiquitin-dependent degradation of HBx protein^18^, and mediates resistance of HCV infection through interacting with CORE by yeast two-hybrid screens[19]and interfering with the viral entry into host cells^20^. Also, it was reported that PLSCR1 forms a trimeric complex with NP and importin α, which inhibits the incorporation of importin β and suppress nuclear importation of NP, thereby inhibiting IAV replication^21^. However, the function of PLSCR1 in vivo has not been clarified. Besides, whether there are other interacting proteins affecting the binding of PLSCR1 and NP will also provide a new mechanism to play an inhibitory role in influenza virus.

The interacting protein of PLSCR1 plays an important role in its antiviral effect. For example, PLSCR1 could interact with the CD4 receptor on the T lymphocyte membrane to inhibit HIV infection and interacts with the angiogenin (ANG )in the nucleus to regulate rRNA transcription^22^. Moreover, PLSCR1 also inhibits vesicular stomatitis virus (VSV) and encephalomyocarditis virus through promoting the secretion of IFN^23^. We used yeast two-hybrid technology to screen whether there are other interacting factors in the complex of PLSCR1 binding to NP, and found immunoglobulin-like receptor 1 (ILDR1) could interact with PLSCR1. ILDR1 is an evolutionally conserved type I transmembrane protein that contains immunoglobulin (Ig)-like domain. ILDR1 and its two paralogs, immunoglobulin-like domain-containing receptor 2(ILDR2) and lipolysis-stimulated lipoprotein receptor(LSR), have been identified as components of tricellular tight junctions (tTJs), specialized structures where the corners of three epithelial cells meet to form a barrier of the cellular sheet^24^. Our previous data showed that ILDR1 interacts with a series of splicing factors and regulates alternative splicing, and it was highly expressed in the lung, the target organ of influenza virus^25^. In this study, we used a combination of knockout mouse models and a series of experiments in vitro demonstrate that the role of PLSCR1 interacting with ILDR1 in regulating IAV infection by promoting the nuclear import of NP.

## Results

### PLSCR1 expression is decreased in mouse lungs after H1N1 SIV infection

**Figure 1:**
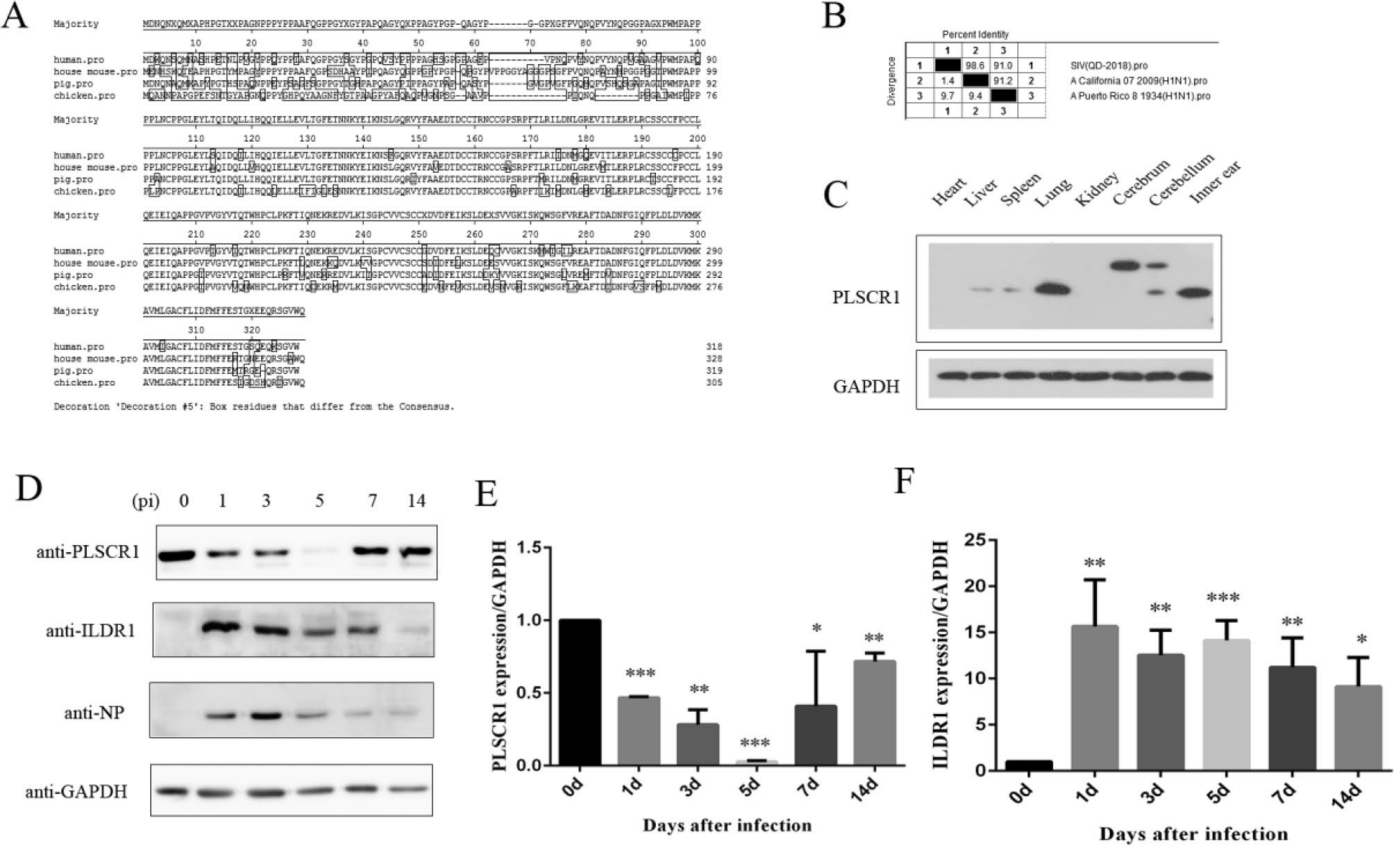
PLSCR1 Expression after H1N1 SIV Infection. (**A**) The similarity of PLSCR1 between different species. (**B**) The identity percentage of nucleoprotein of influenza virus between the isolated strain from the diseased pigs and human. (**C**) Western blots showed that PLSCR1 expression in various tissues of C57BL/6 mice on day 42. (**D**) PLSCR1 and ILDR1protein levels in lung are modulated after swine influenza A virus (SIV) infection. The lungs were dissected from C57BL6/J mice that were either mock- or SIV-infected at multiple days p.i. as indicated. (**E** and **F**) The area of the PLSCR1 and ILDR1 peak, in relation to the standard curve, was determined using ImageJ software (n = 3). Bars represent mean ± s.d. *P < 0.05, **P < 0.01; ***P < 0.001.

PLSCR1 is a calcium ion-binding type II membrane protein, and is highly conserved among different species including human and pigs (Fig. 1A). QD-2018 strain is a type H1N1 SIV strain isolated from pigs in Shandong province of China^26^. This strain has very high homology with human influenza virus isolated in 2009, with the identity of the NP higher than 98.6% (Fig. 1B). This conservation allows us to use SIV in our following investigation of the IAV-associated disease.

We first examined the expression level of PLSCR1 in the tissues of C57BL/6 mice by performing western blot. The results show that PLSCR1 is ubiquitously expressed in various tissues of mice (expect the heart and cerebrum) examined and has relatively high expression level in lung, the target organ of influenza virus (Fig. 1C). We then used 10^3^ pfu H1N1 SIV to infect C57BL/6 J mice intranasally and examined PLSCR1 levels at different time points post infection. The results show that PLSCR1 expression is markedly decreased when examined at 1 day post infection (dpi) (Fig. 1D). PLSCR1 expression continues to decrease to less than 5% at 5 dpi, then gradually returns to the normal level by 14 dpi (Fig. 1D and E). This dynamic change of PLSCR1 expression suggests that PLSCR1 might play an essential role in SIV infection in vivo.

### H1N1 SIV replication is increased in *Plscr1* knockout mice

**Figure 2:**
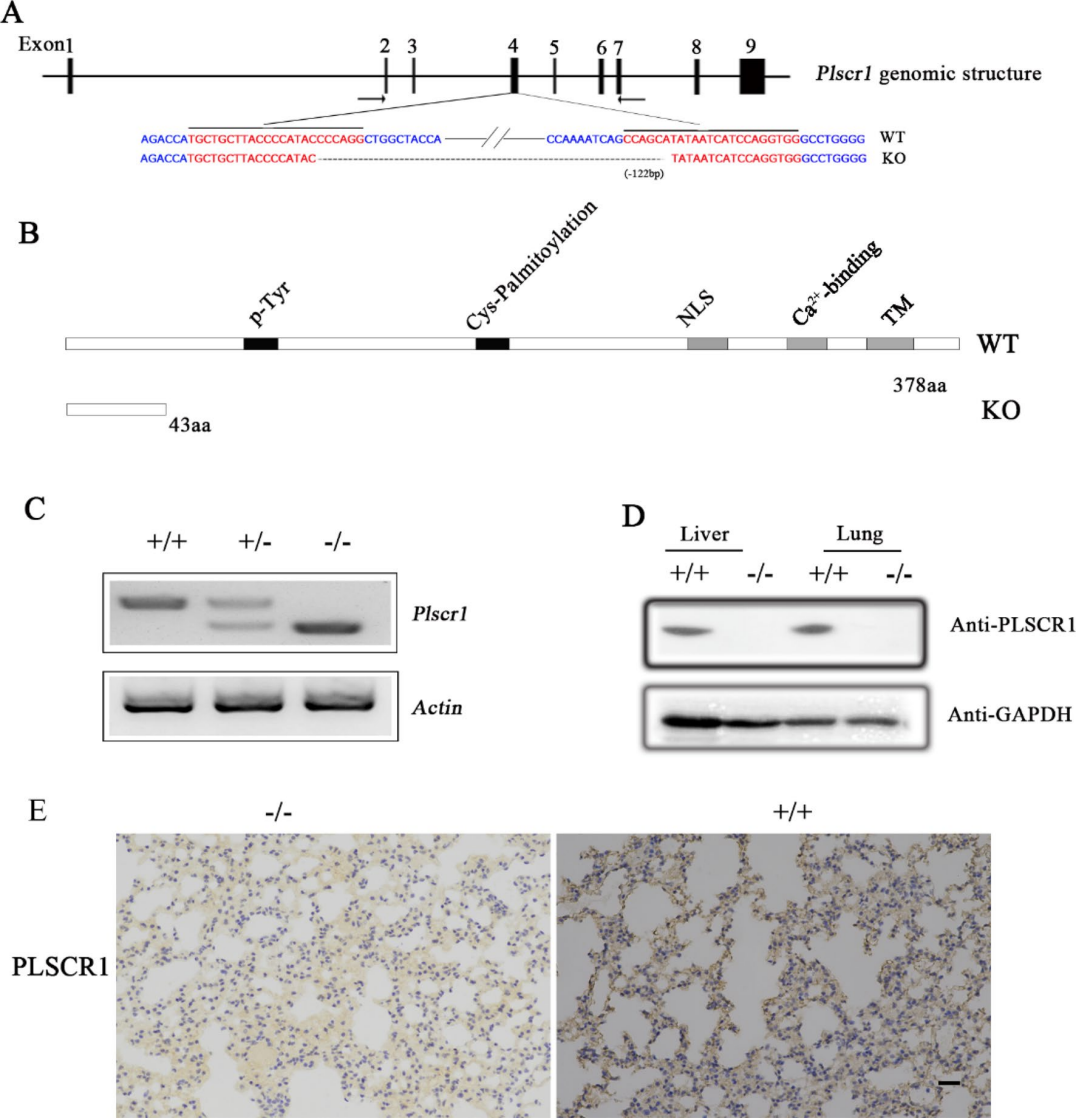
Construction of *Plscr1* knockout mice. (**A**) The schematic drawing of the strategy for *Plscr1* gene disruption. The target sites of clustered regularly interspaced short palindromic repeat (CRISPR)-Cas9 small guide RNAs (sgRNAs) in the *Plscr1* gene are indicated in red, and the deleted region in the *Plscr1* gene of knockout mice is indicated by dashes. The positions of RT-PCR primers are indicated by arrows. (**B**) The schematic drawing of the domain structure of PLSCR1 in wildtype and knockout mice. (**C**) The expression of *Plscr1* mRNA in the tail of P42 mice was determined by RT-PCR. β-actin was included as the internal control. The corresponding size were respectively 636 bp, 514 bp corresponding to wild types and *Plscr1*^*-/-*^ mice. (**D**) Western blot showed the expression in the liver and lung of P42 mice. (**E**) PLSCR1 was detected by immunohistology using rabbit anti-mPLSCR1, visualized with 3,3,-diaminobenzidine, and counterstained with hematoxylin. Scale bar: 20 μm. +/+ :wild type mice; +/− : heterozygous mice; −/−:*Plscr1* knockout mice.

In order to investigate the functions of PLSCR1 during Influenza A virus infection in vivo, we developed *Plscr1* knockout mice using CRISPR/Cas9 genome editing technique. The mouse *Plscr1* gene contains eight exons encoding 378 amino acids, and two sgRNAs were designed to target exon 4 (Fig. 2A). DNA sequencing revealed that a deletion of 122 bp was introduced into exon 4 in *Plscr1* knockout mice, which causes a premature translational stop and gives rise to a potentially truncated protein of 43 amino acids (Fig. 2B). RT-PCR, western blot, and immunohistochemistry results confirm that PLSCR1 expression is indeed disrupted in the homogenous knockout mice (Fig. 2C–E). Interbreeding of *Plscr1*^+/−^ mice gave rise to offspring in the expected Mendelian ratio (23.8% wild-type, 47.6% *Plscr1*^+/−^, and 28.6% *Plscr1*^−/−^; n = 42) with normal viability, suggesting that PLSCR1 is dispensable for general development in mice.

**Figure 3:**
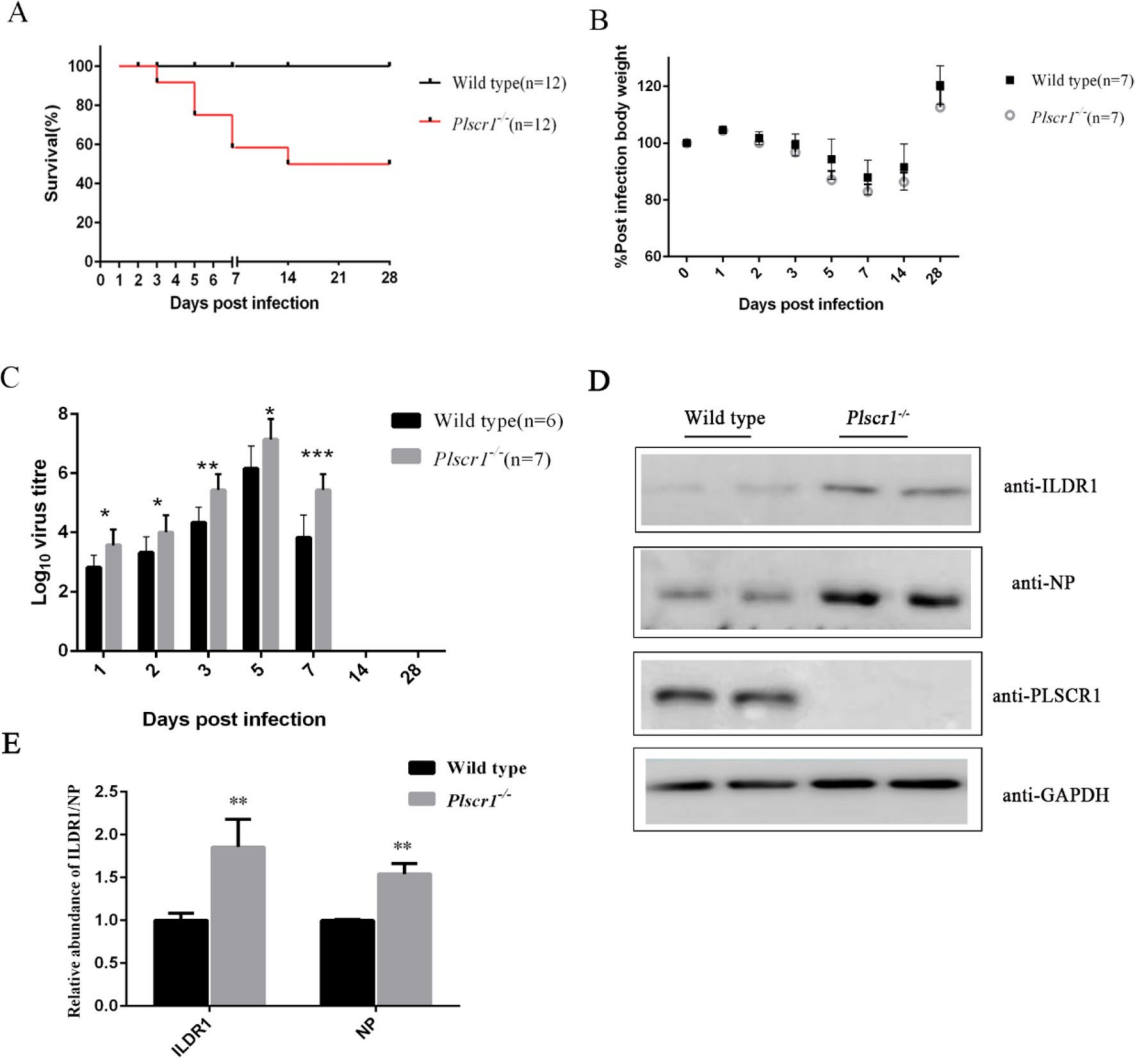
PLSCR1 influences the infection of mice by Swine influenza virus (SIV). (**A**) C57BL/6 J and *Plscr1*^*−*/*−*^ mice were infected intranasally with 10^3^ pfu SIV. Suviral was determined daily and calculated as a percentage of the initial total numbers. (**B**) Mice were weighed daily and the weights represented as a percentage of the starting weight (n = 7 per group). (**C**) Lung tissues were taken at multiple days p.i. as indicated and virus titer determined by plaque assay (n > 6 per group). Data represent the mean value ± SD. Asterisks indicate statistical difference (Unpaired t test, *P < 0.05, **P < 0.01, ***P < 0.001). (**D**) Western blots showed that ILDR1 and NP protein expression in lung of C57BL/6 and *Plscr1*^*−*/*−*^ mice on day 42 after infected with 10^3^ pfu SIV for 3 days. (**E**) Statistical analysis of ILDR1 and NP levels in *Plscr1*^*−*/*−*^ mice. The value for ILDR1 and NP were standardized to the GAPDH level and normalized to the level of ILDR1 and NP in lung of wild type mice. Data are shown as the means SD from three independent experiments (Unpaired t test, *P < 0.05, **P < 0.01, ***P < 0.001).

We then examined whether *Plscr1* knockout affects H1N1 SIV infection. Control and *Plscr1*^−/−^ mice were infected intranasally with 10^3^ pfu H1N1 SIV, and the survival rate, body weight and lung virus load were determined over a 28-day period. The results show that the survival rate of *Plscr1*^−/−^ mice is much lower than that of control mice after SIV infection (Fig. 3A). *Plscr1*^−/−^ mice start to die at 3 dpi, and the survival rate drops to 50% at 28 dpi. Meanwhile, similar body weight was observed between survived *Plscr1*^−/−^ mice and control mice (Fig. 3B). Consistent with the decreased survival rate, higher lung viral load was observed in *Plscr1*^−/−^ mice (Fig. 3C). Titers of SIV increase in the lungs of both genotypes and peak at 5 dpi, then start to decrease and not detectable at 14 and 28 dpi. Virus titers are significantly higher in *Plscr1*^−/−^ mice at all time points examined (Fig. 3C). Consistently, western blotting shows that the level of NP in *Plscr1*^−/−^ mice was significantly higher than that in wild mice at 3 dpi (Fig. 3D and E). Taken together, our present data suggest that H1N1 SIV replication is increased in *Plscr1* knockout mice that leads to lower survival rate.

### ILDR1 is a novel PLSCR1-binding protein

**Figure 4:**
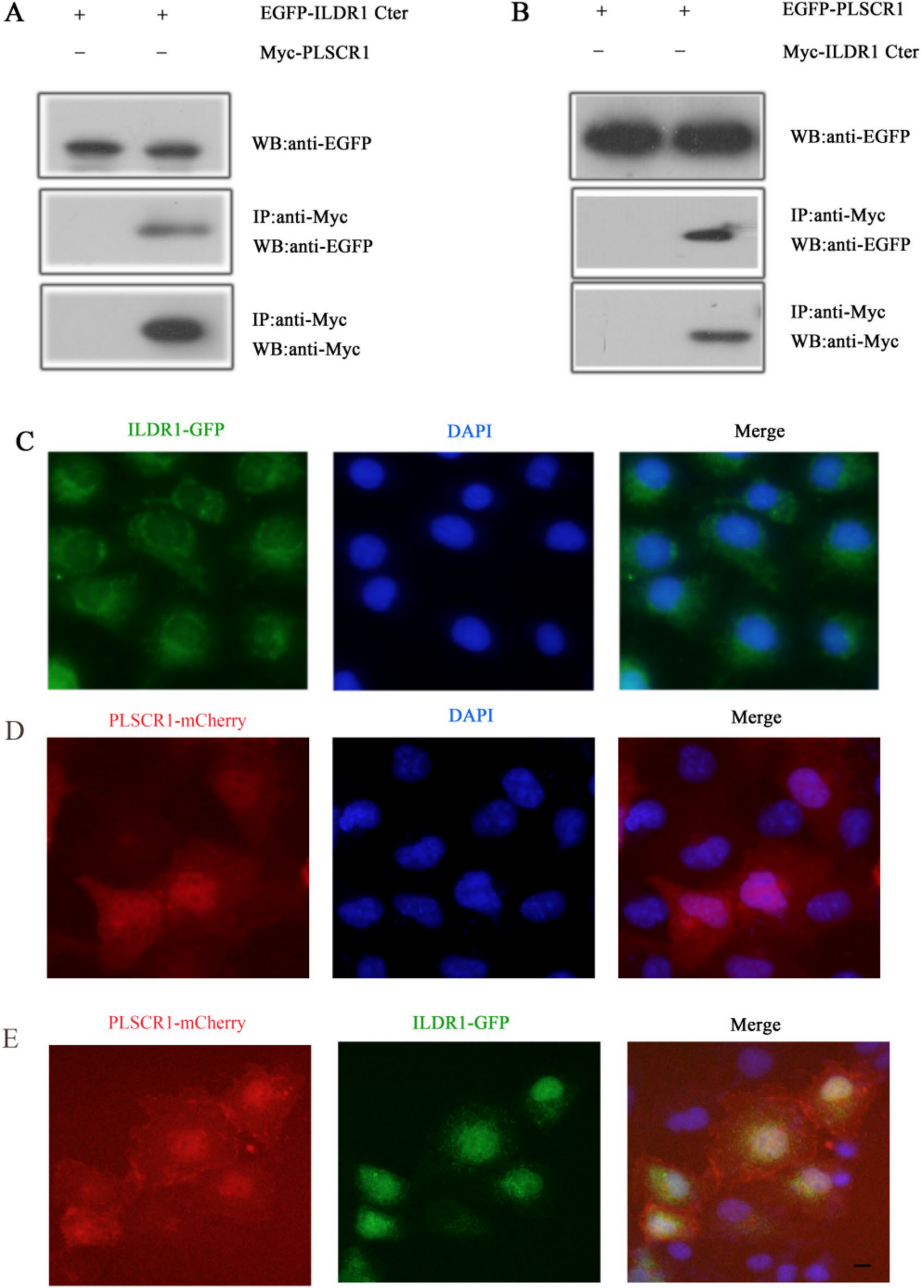
PLSCR1 binds ILDR1. (**A**) Western blots showing that EGFP-tagged cytoplasmic fragment of ILDR1 was co-immunoprecipitated with Myc-tagged PLSCR1. (**B**) Western blots showing that EGFP-taggedPLSCR1 was co-immunoprecipitated with Myc-tagged cytoplasmic fragment of ILDR1. Expression vectors were transfected into HEK293T cells to express epitope-tagged proteins, and cell lysis were subject to immunoprecipitation.5% of total protein was loaded as input. IP indicates antibody used for immunoprecipitation and WB indicates antibody used for detection. (**C**) ILDR1-EGFP localize in the cytoplasm of COS7. (**D**) PLSCR1-mCherry localize both in the nucleus and cytoplasm of COS7. (**E**) However, when PLSCR1-mCherry is present, ILDR1-GFP translocates into the nuclei. Expression vectors were transfected into COS-7 cells to express epitope-tagged proteins. Nuclei were stained with DAPI. Scale bar: 10 μm.

In our previous study, we performed yeast two-hybrid screening of a chicken cDNA library using the C-terminal intracellular domain of ILDR1 (228–553 aa) as bait ^24^. Among the positive clones identified, five clones encode PLSCR1. We then performed co-immunoprecipitation (co-IP) experiments to confirm the interaction between ILDR1 and PLSCR1. The results show that EGFP-tagged mouse ILDR1 cytoplasmic domain could be co-immunoprecipitated together with Myc-tagged PLSCR1 (Fig. 4A). Likewise, EGFP-tagged PLSCR1 could be co-immunoprecipitated together with Myc-tagged ILDR1 cytoplasmic domain (Fig. 4B). ILDR1-EGFP mainly localizes in the cytoplasm in COS-7 cells as reported previously (Fig. 4C) ^25^. In contrast, PLSCR1-mCherry localizes in both cytoplasm and nucleus (Fig. 4D). When cotransfected, ILDR1-EGFP translocates from cytoplasm to nuclei with PLSCR1-Mcherry (Fig. 4E). Our co-IP and co-localization results suggest that ILDR1 is a novel PLSCR1-binding partner.

### ILDR1 promotes H1N1 SIV infection in cultured cells

**Figure 5:**
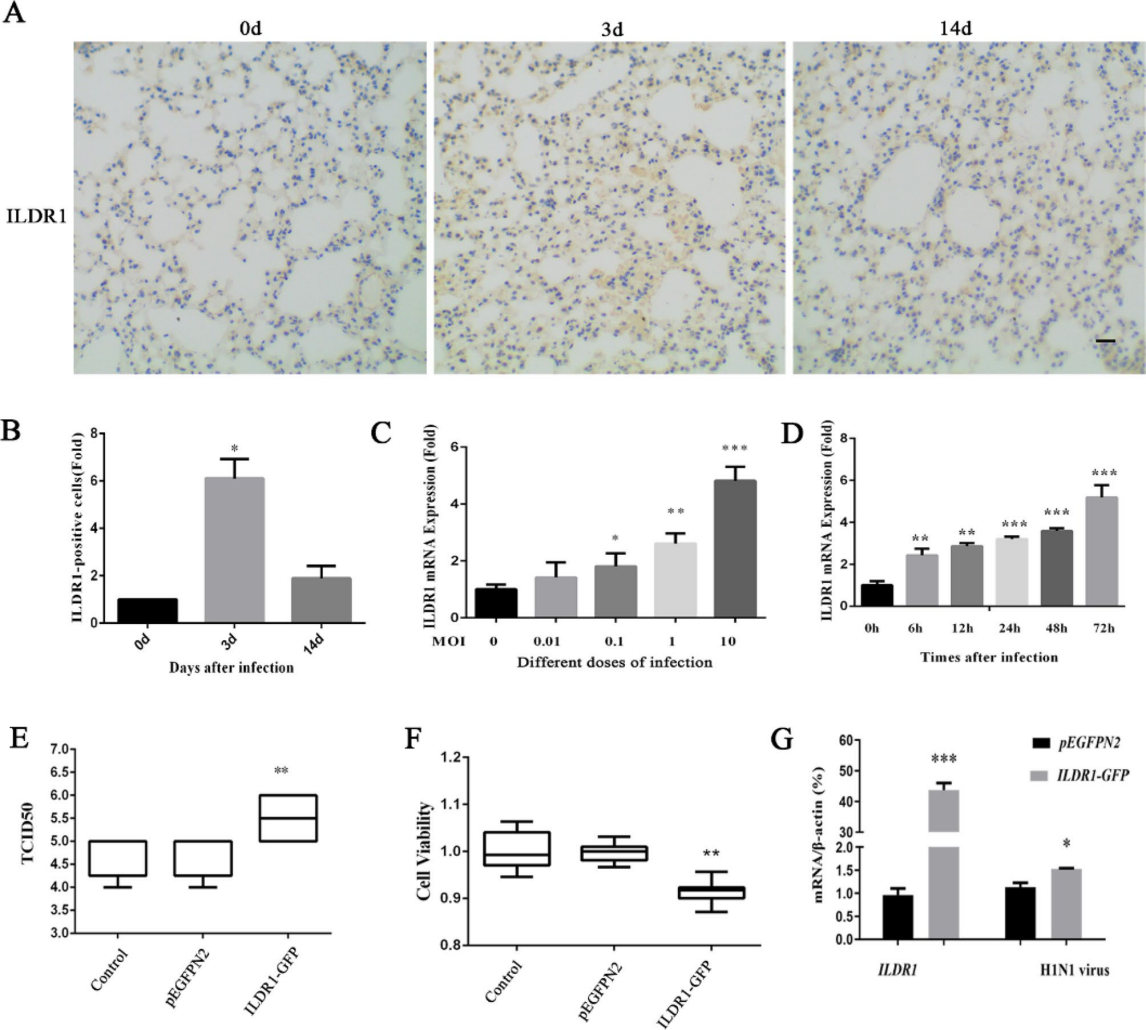
ILDR1 Expression after swine influenza virus infection. (**A**) C57BL/6 J mice were infected intranasally with 10^3^ pfu. SIV. The lungs of mice were taken after infection 0, 3 and14 days for immunohistochemical detection. ILDR1 was detected by immunohistology using rabbit anti-ILDR1, visualized with 3,3,-diaminobenzidine and counterstained with hematoxylin. Scale bar: 20 μm. The expression levels of the ILDR1 after infection relative to that of normal expression were analyzed by areal density (**B**). (**C**) HEK 293 T cells were infected with different doses(MOI = 0,0.01,0.1,1,10) for 48 h, and RNA were extracted, then the expression of *ILDR1* was confirmed by quantitative reverse-transcription PCR. Data represent the mean value ± SD. Asterisks indicate statistical difference (n = 3, Unpaired t test; *P < 0.05, **P < 0.01, ***P < 0.001). (**D**) Expression of *ILDR1* in virus-infected cells at an MOI of 0.1. RNA were extracted at different time points (0 h, 6 h, 12 h, 24 h, 48 h) and subjected to RT-qPCR. Data represent the mean value ± SD. Asterisks indicate statistical difference (n = 3, Unpaired t test; *P < 0.05, **P < 0.01, ***P < 0.001). (**E**) Virus replication in ILDR1-overexpressing HEK293T cells. Cells were transfected with ILDR1-GFP or pEGFPN2 for 24 h, and then infected with SIV at an MOI of 0.1. RNA were extracted at the indicated time points, and virus titers or NP expression were determined by TCID50, CCK-8 (**F**) and RT-qPCR (**G**).

In order to explore the role of ILDR1 in influenza virus infection, we first determined the level of ILDR1 in C57BL/6 J mice after H1N1 SIV infection. Western blot results show that ILDR1 level is significantly increased by more than 15-fold on the first day after infection and then decreases as the viral load decreases (Fig. 1D and F). ILDR1 expression was also examined by performing immunohistology. ILDR1 is weakly expressed in the lungs of mice before infection, while a substantial increase of ILDR1-positive cells was observed at 3 dpi (Fig. 5A and B). The quantitative analysis shows a 3–sixfold (P < 0.05) increase in the percentage area stained in the lungs at 3 dpi. ILDR1 levels was restored to a normal level on 14 dpi (Fig. 5A and B). In addition, ILDR1 levels in *Plscr1*^−/−^ mice were examined after SIV infection at 3 dpi. The results show that ILDR1 expression is up-regulated after viral infection in *Plscr1*^−/−^ mice, suggesting that ILDR1 might play a role in Influenza virus infection (Fig. 3D).

We next analyzed the effect of ILDR1 on SIV infection in HEK 293 T cells. qRT-PCR results reveal that the expression of *ILDR1* mRNA is significantly increased after SIV infection in a dosage- and time-dependent way (Fig. 5C and D). To examine the effect of ILDR1 overexpression, HEK293T cells were transfected with ILDR1-expressing vector or empty vector, followed by infection with SIV at an MOI of 0.1. The results show that SIV TCID_50_ and viral RNA are significantly increased in ILDR1-overexpressing cells (Fig. 5E and G). Meanwhile, the viability of virus-infected cells is significantly decreased in ILDR1-overexpressing cells (Fig. 5F). Taken together, our present data suggest that ILDR1 promotes H1N1 SIV infection.

### ILDR1 competes with NP in binding PLSCR1

**Figure 6:**
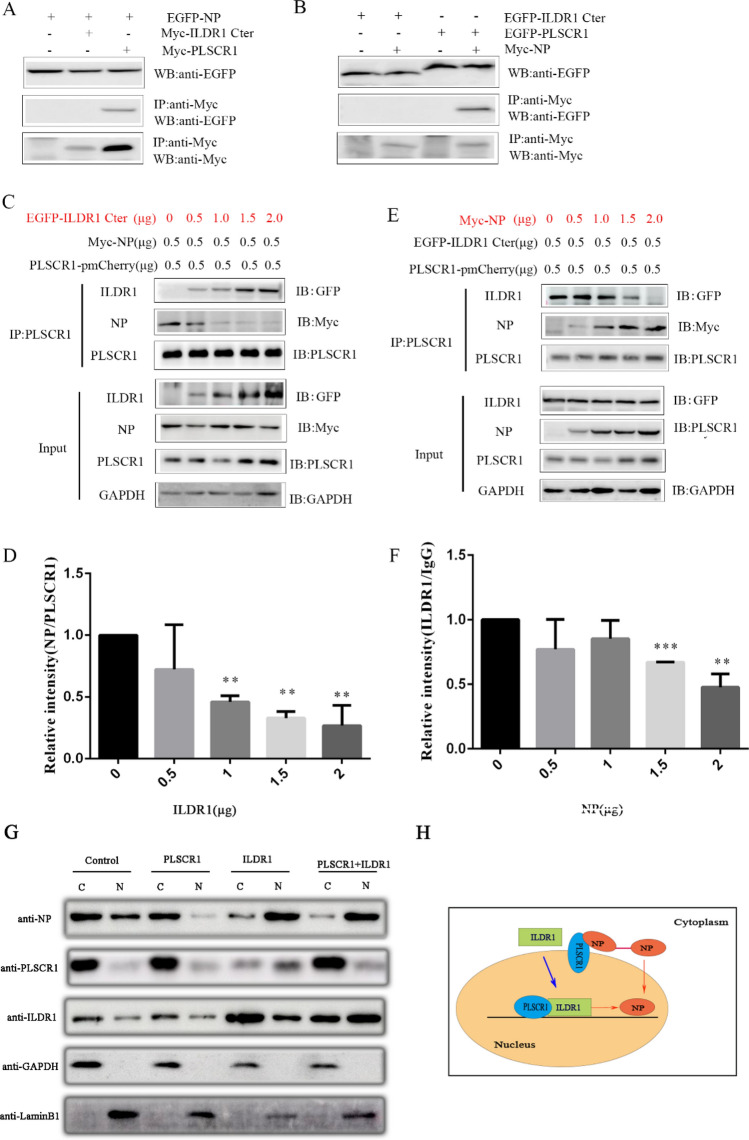
The viral PLSCR1-NP interaction is inhibited by ILDR1. (**A**, **B**) No interaction of ILDR1 with viral NP as determined by the co-IP assays. The interaction between the ILDR1 Cter domain and the NP of SIV was validated using the co-IP assays.HEK293T cells were transfected with ILDR1and NP protein. The Myc-tagged PLSCR1 plasmids were used as positive controls. All cell lysates were prepared at 24 h post transfection and proteins were immunoprecipitated using an anti-Myc mouse MAb, or anti-EGFP rabbit MAb. The immunoprecipitated proteins were analyzed by Western blotting. (**C**) HEK293T cells were transfected with Myc-NP (0.5 μg), PLSCR1-pmCherry (0.5 μg) and different concentrations of EGFP-ILDR1 cytoplasmic fragment (0 μg,0.5 μg,1 μg,1.5 μg,2 μg). The cell lysates were prepared, and proteins were immunoprecipitated using an anti-PLSCR1 rabbit PAb. The expression levels of the ILDR1 and NP protein after immunoprecipitation relative to that of GAPDH were analyzed by densitometry, n = 3 (**D**). (**E**) HEK293T cells were transfected with EGFP-ILDR1 cytoplasmic fragment (0.5 μg), PLSCR1-pmCherry (0.5 μg) and different concentrations of Myc-NP (0 μg,0.5 μg,1 μg,1.5 μg,2 μg). The cell lysates were prepared, and proteins were immunoprecipitated using an anti-PLSCR1 rabbit PAb. The expression levels of the ILDR1 and NP protein after immunoprecipitation relative to that of GAPDH were analyzed by densitometry, n = 3 (**F**). (**G**) Cells were transfected with ILDR1-GFP, PLSCR1-Myc or empty retrovirus-transfected control for 24 h, and then infected with SIV at an MOI of 0.1. At 6 h p.i., the cells were separated into nuclear (**N**) and cytoplasmic fractions (**C**). Each fraction was subjected to western blotting with corresponding antibody for protein detection. (**H**) Model of PLSCR1-NP interaction is competitively by ILDR1. ILDR1 located in the cytoplasm, when in the presence of PLSCR1, ILDR1 and PLSCR1 co-translocate into the nuclei. PLSCR1 could prevent the nuclear import of NP protein and ILDR1 could bind to PLSCR1 competitively with NP. So in the nuclei, the viral PLSCR1-NP interaction is inhibited by ILDR1.

We transfected either ILDR1-Cter or PLSCR1 together with NP protein in HEK 293 T cells and examined their potential interaction by performing co-IP experiments. The results show that Myc-tagged PLSCR1 but not ILDR1-Cter is co-immunoprecipitated with EGFP-tagged NP protein (Fig. 6A). Likewise, EGFP-tagged PLSCR1 but not ILDR1-Cter is co-immunoprecipitated with Myc-tagged NP (Fig. 6B). We then moved on to examine whether ILDR1 and NP could interact with PLSCR1 simultaneously or compete with each other. We examined the interaction between NP and PLSCR1 in the presence of ILDR1 of increasing amounts. The results show that when the amount of ILDR1 is increased, the level of NP bound to PLSCR1 is decreased (Fig. 6C and D). Similar results were obtained when increasing amounts of NP was used (Fig. 6E and F).

In order to further verify that ILDR1 and PLSCR1 will affect the nuclear importation of NP, a cell fractionation experiment was conducted. Cells were transfected with ILDR1-GFP, PLSCR1-Myc or empty plasmid—transfected control for 24 h, and then infected with SIV at a MOI of 0.1. At 6 h p.i., the cells were separated into nuclear (N) and cytoplasmic fractions (C), then subjected to western blotting. The results show that, GAPDH and LaminB1 were only detected in the cytoplasm and nucleus, respectively. Also we found that NP was primarily detected in the cytoplasm and was only weakly detected in the nucleus while PLSCR1 was overexpressed as reported previously ^21^. In contrast, when we overexpressed ILDR1, it will promote the expression of NP protein into the nucleus. When ILDR1 and PLSCR1 were co-transfected, a considerable amount of NP was detected in both the nucleus and the cytoplasm (Fig. 6G). Taken together, our present data suggest that ILDR1 and NP compete with each other in binding PLSCR1(Fig. 6H).

## Discussion

Influenza A virus has caused several outbreaks of influenza in human history, including the 2009 H1N1 Influenza pandemics. As a single-strand negative-strand, segmented RNA virus, its high mutation rate and gene rearrangement properties can continuously produce new virus subtypes, imposing a long-term threat to human and animal health. Swine plays an important role in the recombination of influenza virus because of the characteristics of the receptors^3^. In 2009, the H1N1pdm09 IV pandemic broke out in the world, and researchers found that many of its gene fragments are derived from SIV^1^. Between 2011 and 2018, a predominant emergent Eurasian avian-like (EA) reassortant genotype 4 (G4) virus in pigs had pdm/09 and TR-derived internal genes and showed increasing human infectivity^2^. In addition, the H1N1 subtype SIV has always maintained a high prevalence rate in the pig herd ^27^, and has been involved in the recombination of a variety of new viruses during its evolution ^28^. Therefore, our work is of great significance for exploring the pathogenic mechanism of SIV. The experimental strain used in the present work was collected from a sick pig farm. It showed homology of more than 90% with the 2009 human epidemic strain and mouse-adapted A/Puerto Rico/8/34 stain. Also, the virus could infect mice and the half infection amount of the virus was10^–4.48^ by the Reed-Muench method. It thus models IAV-associated disease in humans and also allows for analysis over an extended time course.

Influenza A virus ribonucleoprotein (vRNP) complex is the structural basis of viral RNA transcription and replication, and plays an important role in the process of virus infection^29^. During the early stage of viral infection, the vRNP complex is released into the cytoplasm of the host cell via endocytosis and demembrane, and then enters the nucleus with the help of nuclear localization signals and transport proteins^30^. To date, several host proteins have been reported to be potential binding partners of the vRNP complex, and regulate viral infection through various mechanisms^5^. In our experiment, we established *Plscr1* knockout mice and found that it is indeed more susceptible to SIV. By examining the survival rate, body weight and lung virus load of the mice, we presented in vivo evidence for the first time that PLSCR1 is important for inhibiting influenza virus replication, which was consistent with the report of PLSCR1 negatively regulates virus replication by interacting with NP in the cytoplasm and preventing its nuclear import in vitro^20^. We also found that PLSCR1 is widely distributed in various tissues of mice (except heart and brain). Among these, PLSCR1 is highly expressed in the lung, the target organ of influenza virus. At the same time, we observed the higher bands of PLSCR1 in the cerebrum and cerebellum (approximately 55 kD) were initially detected, then we tested the expression of PLSCR1 in wild mice and *Plscr1*^−/−^ mice by western blot, and found that this band still exists, so it is speculated that it may be a non-specific band. This results have also been verified by Sahu SK in human tissue^31^.

The interaction protein of PLSCR1 plays an important role in its antiviral effect, so whether there are other proteins affecting the interaction between PLSCR1 and NP during influenza virus infection. We identified ILDR1 as a novel PLSCR1-binding partner by yeast two-hybrid. ILDR1 is a putative type I transmembrane protein containing an immunoglobulin (Ig)-like domain^32^. ILDR1 has been shown to mediate fat-stimulated cholecystokinin (CCK) secretion^33^ and play an important role in regulating the integration of tTJs^23^. *Ildr1* knockout mice show profound hearing loss accompanied with tTJs destruction in the inner ear^34,35^ and polyuria due to renal concentrating defects in kidneys^36^. In our previous study, we detected high expression of ILDR1 in lungs of mice^25^. However, its function in the lungs and whether it participates in disease infection has not been reported so far. Therefore, we observed the role of ILDR1 in influenza virus infection. We first determined the level of ILDR1 after H1N1 SIV infection by western blot and immunohistochemistry. The results showed that ILDR1 levels significantly increased by more than 15-fold on the first day after infection and it decreased as the viral load decreased. The same results were obtained using *Plscr1* knockout mice. Moreover, we examined the effect of ILDR1 overexpression on virus infection, and confirmed that virus replication was significantly promoted and the viability of virus-infected cells is decreased, suggesting that ILDR1 might play a role in influenza virus replication. Also, its homologous protein ILDR2 have been extensively studied on immunomodulatory effect in recent years. ILDR2, a new B7 family protein, has been confirmed expressed in immune cells and inflammatory tissues to suppress the activity of T cells^37^. ILDR2-Fc regulates the function of immunity in the treatment of autoimmune diseases by regulating the stability of the immune internal environment and rebuilding the balance of immune tolerance^38^. However, the expression of this family member in virus infection and its pathophysiological significance have not been reported so far. Further studies were needed to determine exactly how the ILDR1 protein family play in influenza virus, which may be a new target of influenza virus or a new direction provided for influenza virus research.

Given that PLSCR1 can inhibit influenza virus replication by binding to the NP protein^21^, and ILDR1 could interact with PLSCR1, so the role of ILDR1 on the PLSCR1-NP complex in influenza virus deserves further study. The fact that ILDR1 could not directly interact with NP while PLSCR1 could interact with NP protein as previously reported. However, when the amount of ILDR1/PLSCR1 is increased, the level of NP bound to PLSCR1/ ILDR1 is decreased, which indicating that ILDR1 and have a certain competitive effect on the binding with NP. Additionally, ILDR1 will promote the expression of NP protein into the nucleus when overexpressed by cell fractionation experiment. The redundant PLSCR1 binds to the NP protein, which may facilitate the entry of the NP protein into the nucleus, thereby promoting the replication of the virus. However, the precise regions of the PLSCR1 binding to ILDR1 and NP should be investigated in the future to understand this competitive interaction affecting the functions of the viral RNP complex.

In conclusion, this study identified the ILDR1 promotes SIV replication through interacting with PLSCR1. PLSCR1 was shown to inhibit influenza virus replication in vivo and could interact with ILDR1. The expression level of ILDR1 is increased after virus infection and as an antiviral suppressor to inhibit viral replication. Also, ILDR1 could not directly interact with virus NP protein, but could combine with PLSCR1 competitively. This study suggests the existence of a previously unknown pathway in regulating SIV infection, which sheds light on SIV prevention and/or treatment.

## Materials and methods

### Animals

Generation of *Plscr1* Knockout mice were generated using the clustered regularly interspaced short palindromic repeat (CRISPR)-associated Cas9 nuclease (CRISPR/Cas9) genome editing technique as previously described^39^. Briefly, C57BL/6 female mice (7–8 weeks old) were superovulated by intraperitoneally injecting pregnant mare serum gonadotropin (PMSG) and human chorionic gonadotrophin (hCG) and then mated to C57BL/6 male mice. The fertilized embryos (zygotes) were collected from the oviducts, and mixed Cas9 mRNA (50 ng/µl) and small guide RNA (sgRNA; 25 ng/µl) were injected into the cytoplasm of zygotes with visible pronuclei in Chatot-Ziomek-Bavister (CZB) medium. The injected zygotes were then cultured in Quinn’s Advantage cleavage medium (in vitro Fertilization, Inc.) for 24 h, at which time 18–20 2-cell–stage embryos were transferred into the oviduct of a pseudopregnant ICR female mouse at 0.5 day post coitus (dpc). The accession numbers of the *Plscr1* cDNAs used to design sgRNA is NM_011636.2. To determine the nucleotide sequence of mutated alleles, genomic DNA of F0 mice was amplified using the following primers: forward, 5′-GGTGATCTCGATTCAGGGGT-3′, reverse, 5′-GGGGTTACTCGACCCTAAAA-3′. DNA sequencing was then performed after TA cloning into plasmid pMD19T. In order to obtain F1 knockout mice, F0 mice were crossed with C57BL/6 mice and newborns were examined by Sanger sequencing. All animal experiments were approved by the ethics committee of Shandong Academy of Agricultural Science. All methods were performed in accordance with the relevant guidelines and regulations. At the time of sacrifice, animals were euthanized with an overdose of isoflurane to minimize suffering followed by decapitation.

### Virus

SIV (QD-2018 strain) was a H1N1 strain that isolated and obtained from the diseased pigs in Shandong province of China, which can be used as one of representative strains for the analysis of variant strains. The virus was continuously passaged on MDCK cells and virus titer was as high as 10^–4^.^48^ TCID_50_/mL.

### Cell culture, transfection

HEK 293 T(Human embryonic kidney cells, CC-Y1010), COS7(Monkey Kidney Cell, provided by Pro. Zhigang Xu) and A549 cells (Human lung cancer cells, ATCC CCL-185) were maintained with 10% FBS DMEM medium at 37 °C under 5% CO_2_, and transfected with expression vectors or siRNAs using Lipofectamine 2000 Transfection Reagent (Thermofisher) according to the manufacturer’s instructions.

### Plasmid construction

The cDNAs encoding the mouse *Ildr1* (NM_001285788.1) and *Plscr1* (NM_011636.2) were cloned into pmCherry-N1, pEGFP-C2, and pMyc-C2 (modified pEGFP-C2 with EGFP-coding sequence replaced by Myc-coding sequence) as we have reported. All the constructs were verified by Sanger sequencing.

### Yeast two-hybrid screening

Yeast two-hybrid screening was performed as described previously ^40^. Briefly, yeast strain AH109 (Clontech, Mountain View, CA, USA) was sequentially transformed with the baitplasmid and a chicken cochlear cDNA library in HybriZAP pAD-GAL4 vector50. HIS3 was used as the reporter

gene for the screening in presence of 2.5 mM 3-amino-1,2,4-triazole (3-AT). Positive colonies were further tested for activation of two other reporter genes, ADE2 and lacZ. Then the pAD-GAL4 prey vectors in triple-positive colonies were recovered, and cDNA inserts were determined by Sanger sequencing.

### Western blot

Cultured cells were transfected with expression vectors as described above or siRNAs synthesized by the Sigma-Aldrich company, then protein was resuspended with RIPA cell lysis containing 1 mM PMSF (Beyotime; Shanghai, China). After centrifuging at 4℃ for 20 min, the supernatant was analyzed by western blot. Protein samples were resolved by 10% SDS-PAGE, then transferred to a PVDF membrane. After blocking with 5% BSA buffer for 1 h, the membrane was cut prior to hybridisation with antibodies. They were incubated with Rabbit Polyclonal-PLSCR1 Polyclonal Antibody (Proteintech, Cat#11582–1-AP,1:1000 diluted), Rabbit Polyclonal-Anti-ILDR1 antibody (Abcam, Cat#ab89847,1:1000 diluted), Rabbit Polyclonal-Anti-H1N1 Influenza A virus Nucleocapsid protein antibody(Abcam, Cat#ab104870,1:1500 diluted), rabbit anti-GAPDH antibody(Abcam, Cat#ab181602, 1:5000 diluted), rabbit anti-LaminB1 antibody(Abways, Cat#AB0054, 1:3000 diluted) at 4℃ over night, followed by incubation with goat anti-mouse secondary antibody or goat anti-rabbit secondary (Cell Signaling Technology, Danvers, MA) at room tempreture for two hours. The 26,616-PageRuler Prestained Protein Ladder was provided by Thermo Fisher Scientific. The signals were detected with the ECL system (ImageQuant LAS 500, GE, USA) or exposed in dark room. The area of the related proteins peak, in relation to the standard curve, was determined using ImageJ software.

### RNA extraction, RT-PCR and Quantitative real-time PCR

Total RNA was isolated from virus-infected cells or mouse tissues which frozen-thawed for three times and cDNA was carried out by reverse transcription (RT) following the kit instructions (TaKaRa Bio Inc., Dalian, China). The expression of gene or virus was analyzed by quantitative real-time PCR (SYBR® Premix Ex TaqTM system, Takara). The primers used were as follows: *Ildr1* forward primer, CCGGCGGCTGATGAAGAAAGACTC, reverse primer, AGGGCAGCAACAGCGGGTAGGA; *Plscr1* forward primer, GTGGGGCGTC TAGACCTTTC, reverse primer, CCAGGCATCACAGGTGAGTT; H1N1 forward primer, ACAGAAGTTATAAGAATGA, reverse primer, TGTCTCCGAAGAAAT AAGA;β-actin forward primer, ACGGCCAGGTCATCACTATTG, reverse primer, AGGGGCCGGACTCATCGTA. PCR reaction system and procedures were refered to Premix Taq kit instructions (Takara). PCR reaction sets were adjusted between 24 and 36 cycles, and annealing temperatures were adjusted between 55 and 60 °C. The PCR products were separated by electrophoresis on agarose gel.

Quantitative real-time PCR was carried out using SYBR® Premix Ex TaqTM system (Perfect Real Time, Takara). The primers and templates were the same as that used in RT-PCR. Amplifiation and detection were run in a Roche 480 Sequence Detection System with an initial cycle of 95 °C for 10 s followed by 40 cycles of 95 °C for 5 s, 62 °C for 10 s and 72 °C for 5 s. All PCR reactions were performed in triplicate. Fold change in gene expression level was calculated using the 2^-ΔΔct^ method and all PCR reactions were performed in triplicate.

### Immunofluorescence assay

Infected cells or transfected cells with GFP- or mCherry-tagged proteins growing on Gelatin-coated glass cover slips were fixed with 4% paraformaldehyde (PFA) in PBS for 15 min and blocked with PBT1 buffer (0.1%Triton X-100, 1% BSA, 5% heat-inactivated goat serum in PBS, pH 7.3) for 30 min. Cells were incubated overnight at 4 °C with corresponding antibody diluted in PBT1 then washed twice with PBS for 10 min. And cells were incubated with FITC-conjugated secondary antibody diluted in PBT2 (0.1% Triton X-100, 0.1% BSA in PBS) for 1 h, followed by washing with PBS three times for 10 min. For nuclei staining, cells were incubated with DAPI (Solarbio Life Sciences) for 15 min, followed by three 10 min PBS washes, then mounted in 50% glycerol/PBS. The cells were imaged with an inverted fluorescence microscope (TE200, Nikon).

### Immunohistochemistry

The tissues were fixed in 10% formalin solution, embedded in paraffin, sectioned to 4 μm thicknesses and dewaxed the paraffin sections to water. Then the sections were placed in a retrieval box filled with EDTA antigen retrieval buffer (PH 8.0) in a microwave oven for antigen retrieval. After that, the sections were blocked with 5% BSA for 30 min at room temperature. The section were incubated with anti-PLSCR1antibody (1:50) overnight at 4 °C. After a brief wash, the secondary antibody were used for detection at room temperature for 50 min and DAPI dye solution was added for 10 min in the dark. The images were taken using a light microscopy (Nikon Eclipse C1). For the controls, no antibody was added to the samples.

### Cell fractionation

The PLSCR1-overexpressing or empty retrovirus-transfected control A549 cells grown in 6-well plates were infected with WSN virus at an MOI of 5. At 6 h p.i., the cells were separated into nuclear and cytoplasmic fractions by using Minute Nuclear and Cytoplasmic Extraction Reagents (SC-003, Invent) according to the manufacturer's procedure. The amount of NP, ILDR1 and PLSCR1 in each fraction were determined by western blotting with a rabbit anti-NP pAb, a rabbit anti-ILDR1 pAb and a rabbit anti-PLSCR1 pAb, respectively. LaminB1 and GAPDH, nuclear and cytoplasmic fraction markers, respectively, were detected by western blotting with a rabbit anti-GAPDH pAb and a rabbit anti-LaminB1 pAb, respectively.

### Co-immunoprecipitation (co-IP)

HEK293T cells were transfected with expression vectors as described above, then washed twice with PBS 24 h after transfection and resuspended in ice-cold lysis buffer containing 150 mM NaCl, 50 mM Tris at pH 7.5, 0.1% Triton X-100, and 1 × cocktail (Roche, Basel, Switzerland). After centrifuging at 4 °C for 20 min, the supernatant was collected and incubated with immobilized anti-Myc or anti-EGFP antibody at 4 °C overnight. Immunoprecipitated proteins were washed three times with 300 mM lysis buffer and then analyzed by western blot.

### Statistical analyses

All data were expressed as means ± standard deviation (SD), and an independent-sample *t*-test was used to evaluate data using Graph Prism software. *P < 0.05, **P < 0.01, ***P < 0.001.

### ARRIVE guidelines statement

The ARRIVE Guidelines have been adopted.

## Supplementary Information

Supplementary Information.

## Data Availability

The datasets generated and analysed during the current study are available from the corresponding author (wujiaqiang2000@sina.com) on reasonable request.
