# Supplementary figures and images for: ILDR1 promotes influenza A virus replication through binding to PLSCR1
Source: Sci Rep. 2022 May 20;12:8515. doi: 10.1038/s41598-022-12598-3 (PMC9122930; doi:10.1038/s41598-022-12598-3)

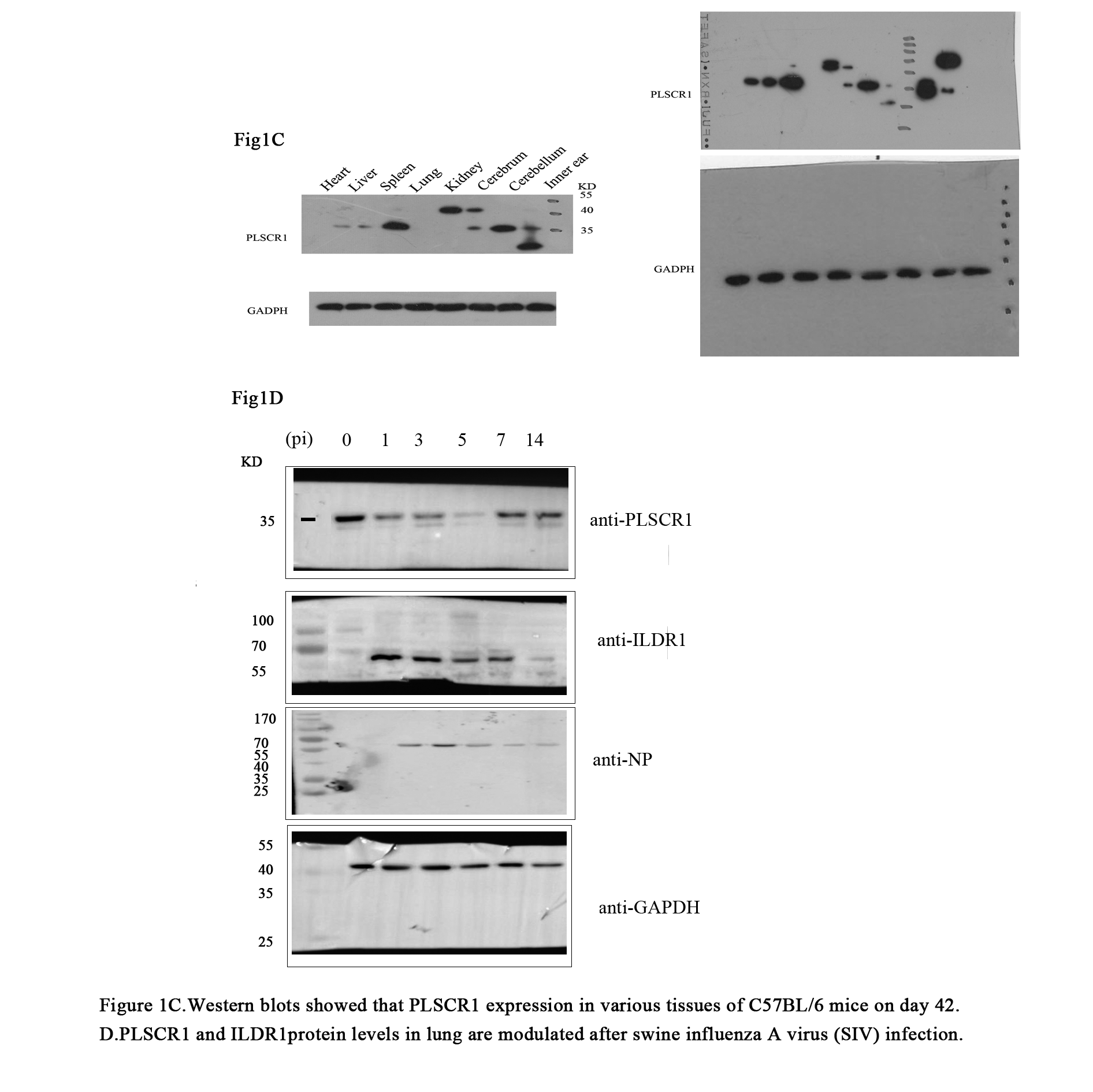

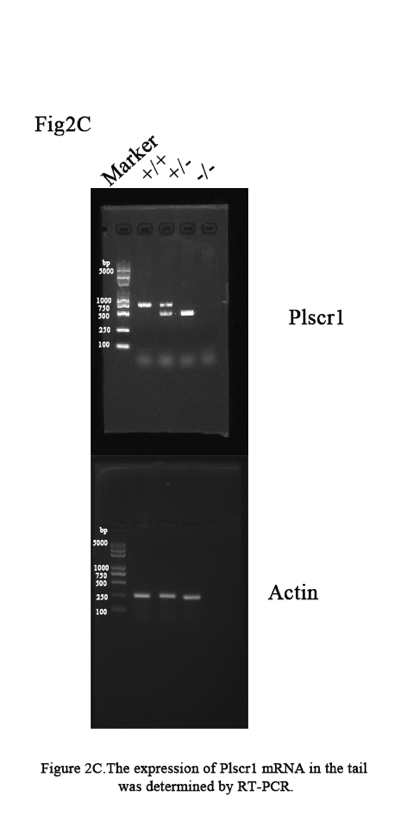

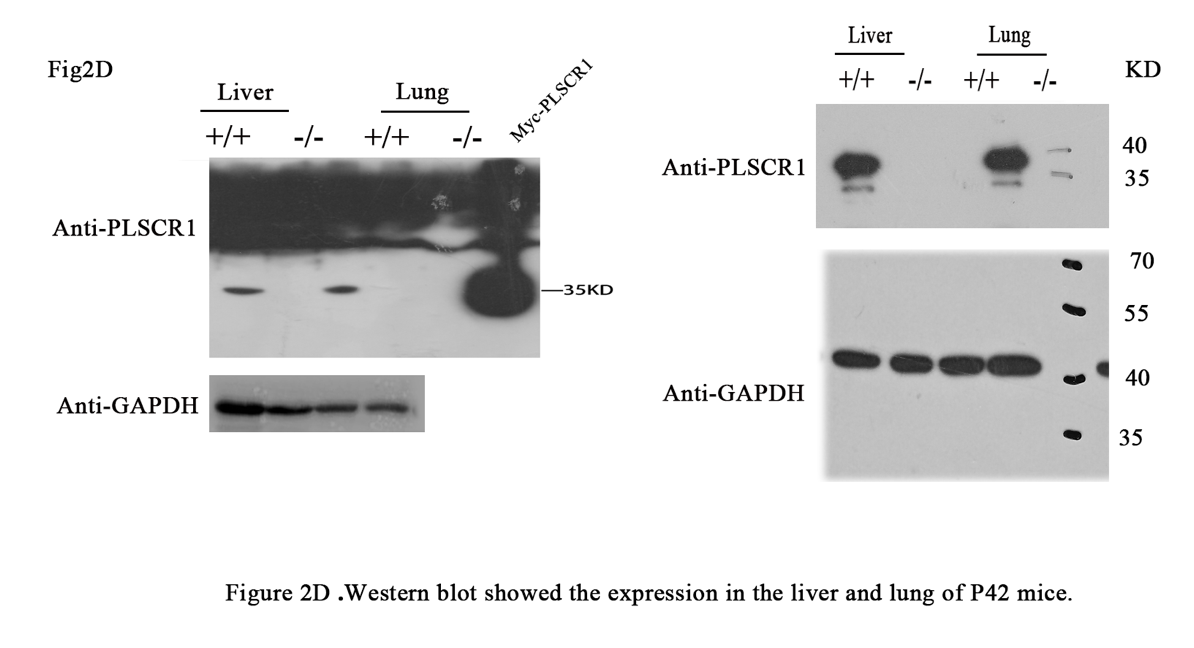


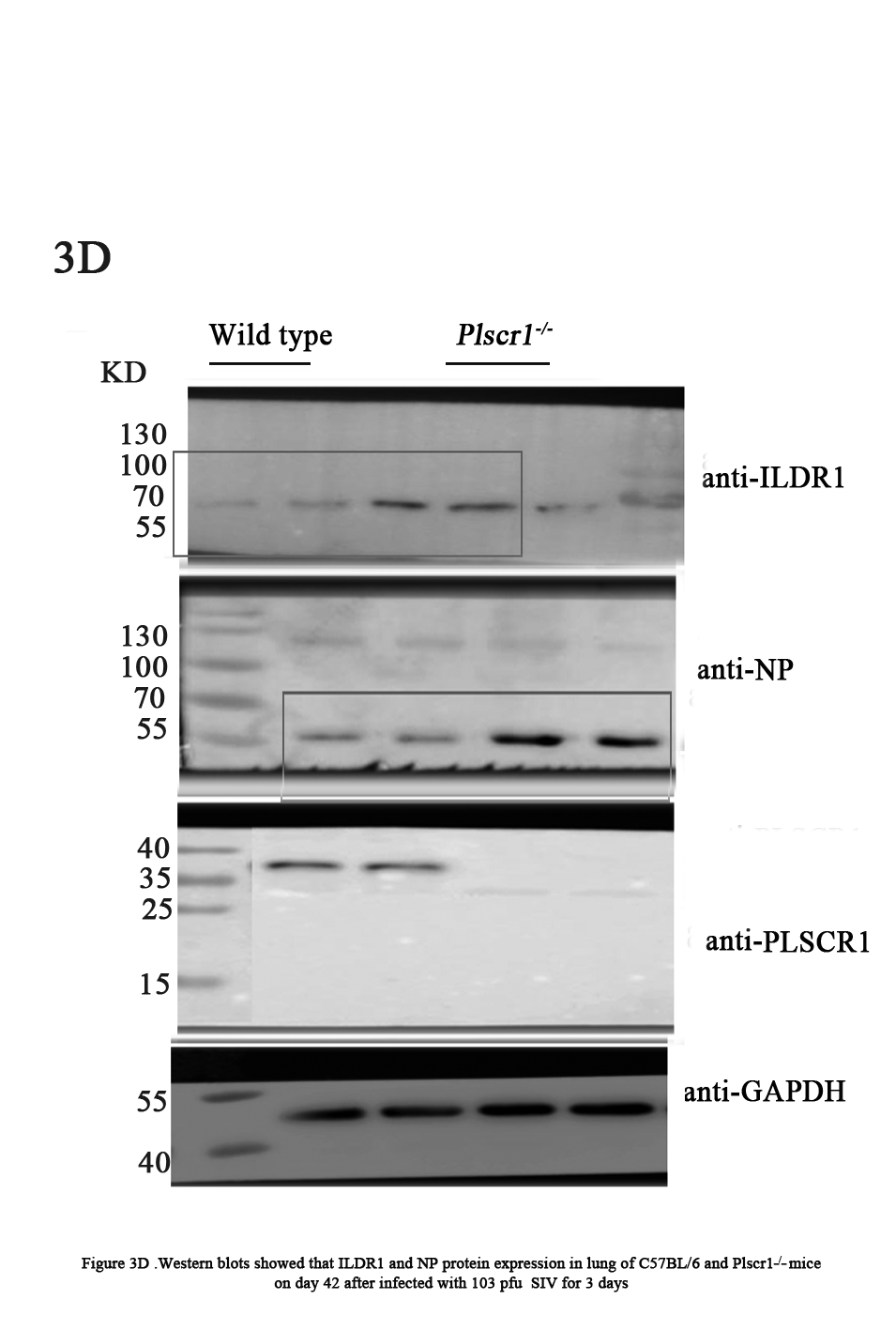

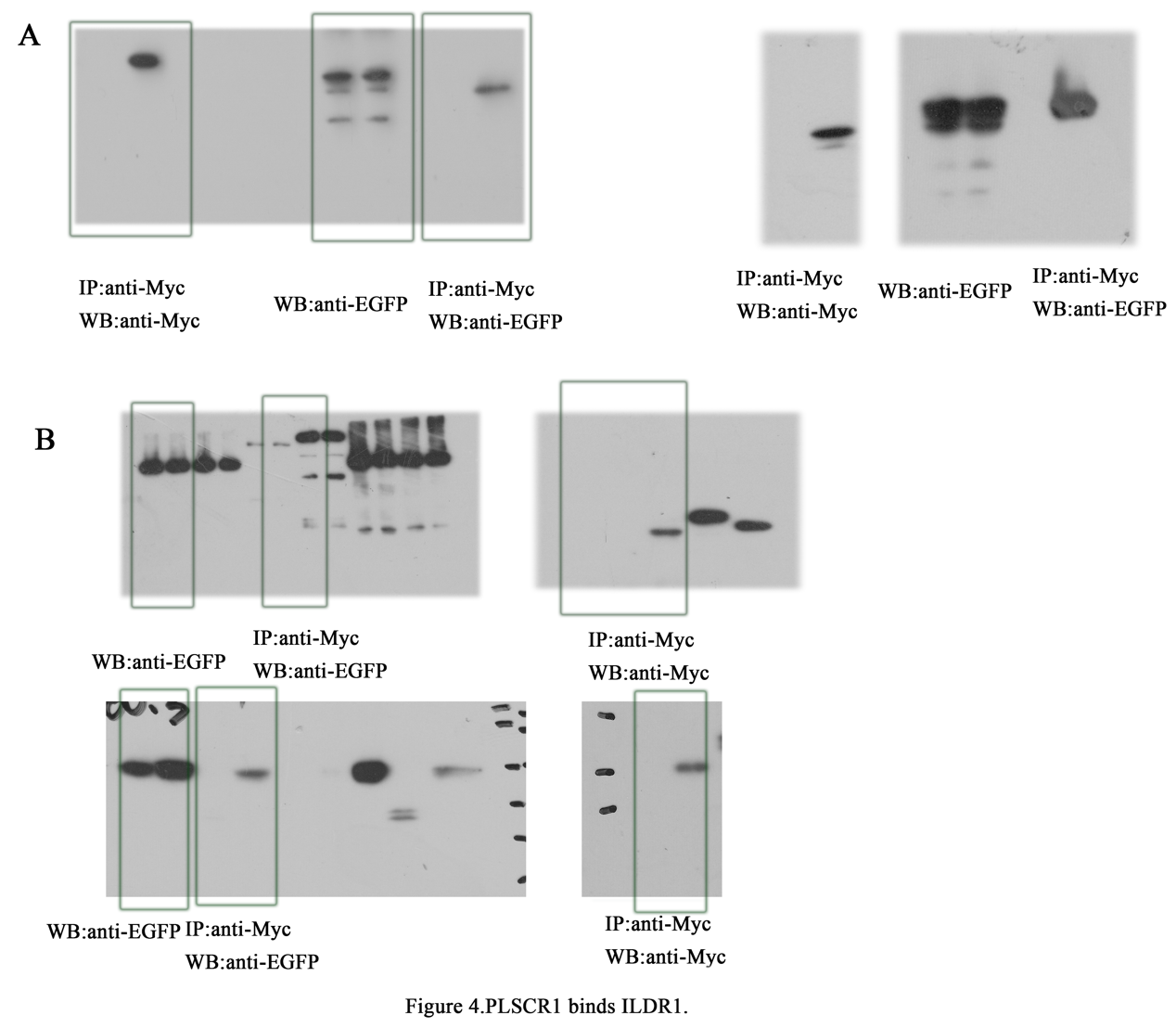


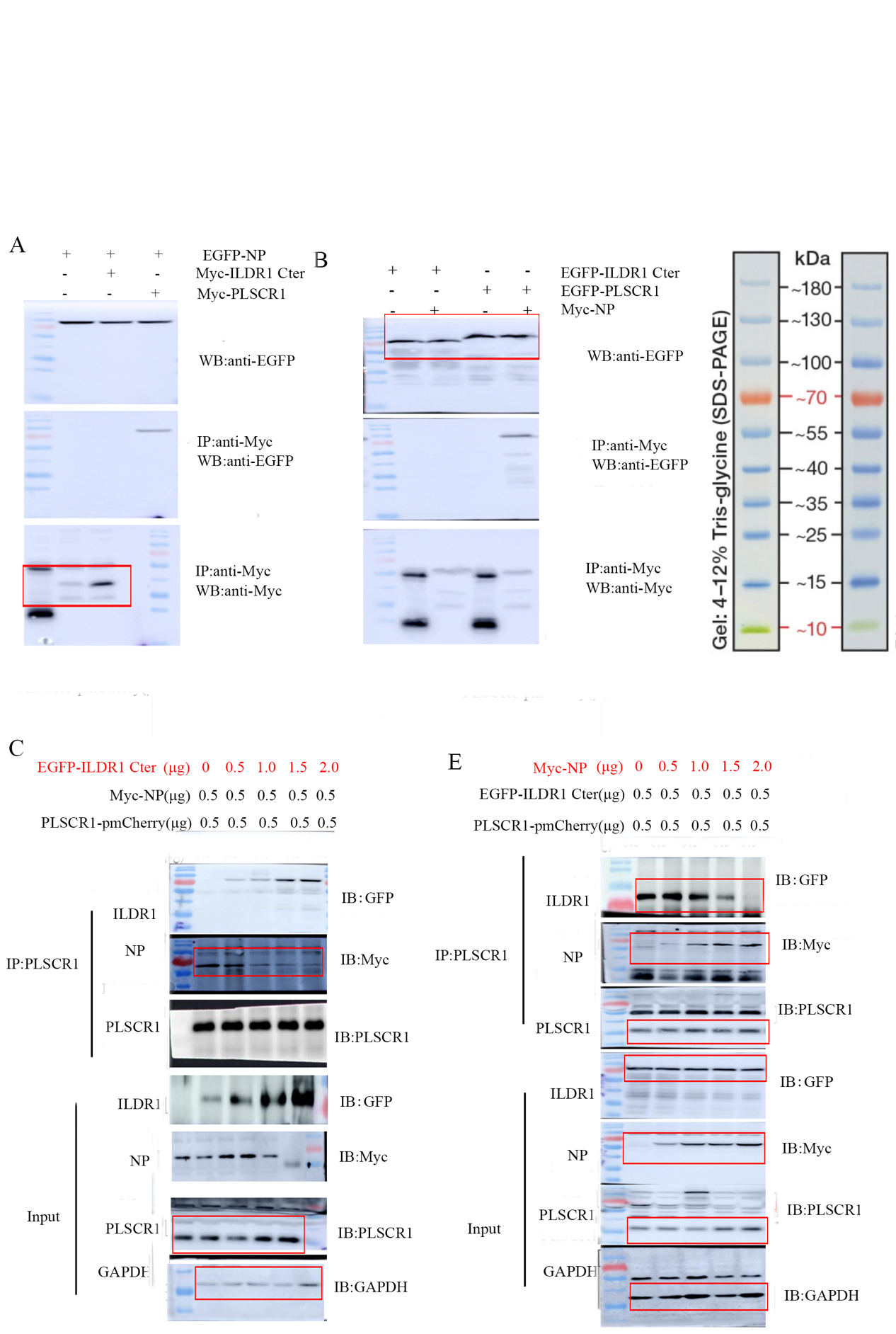

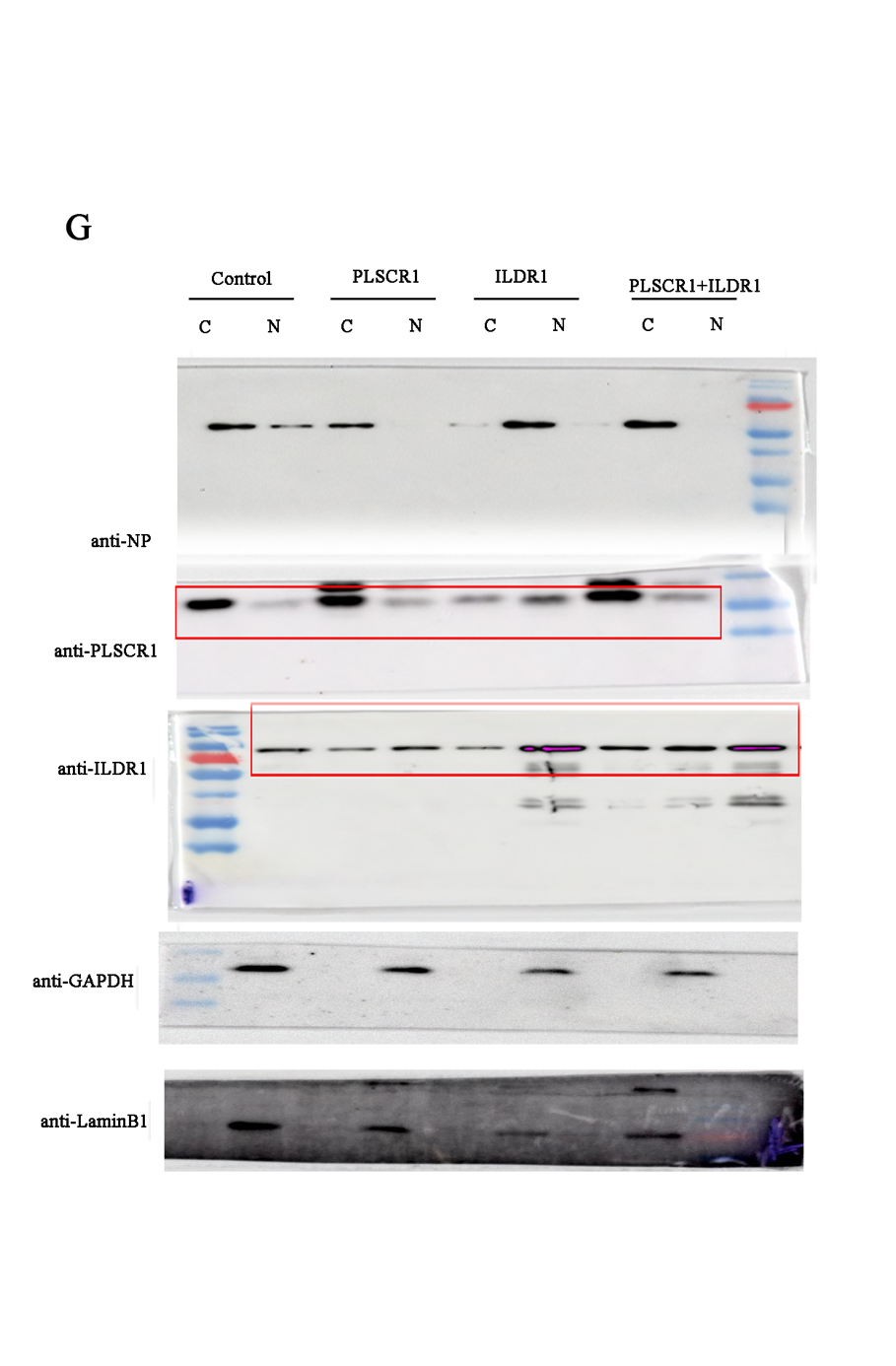

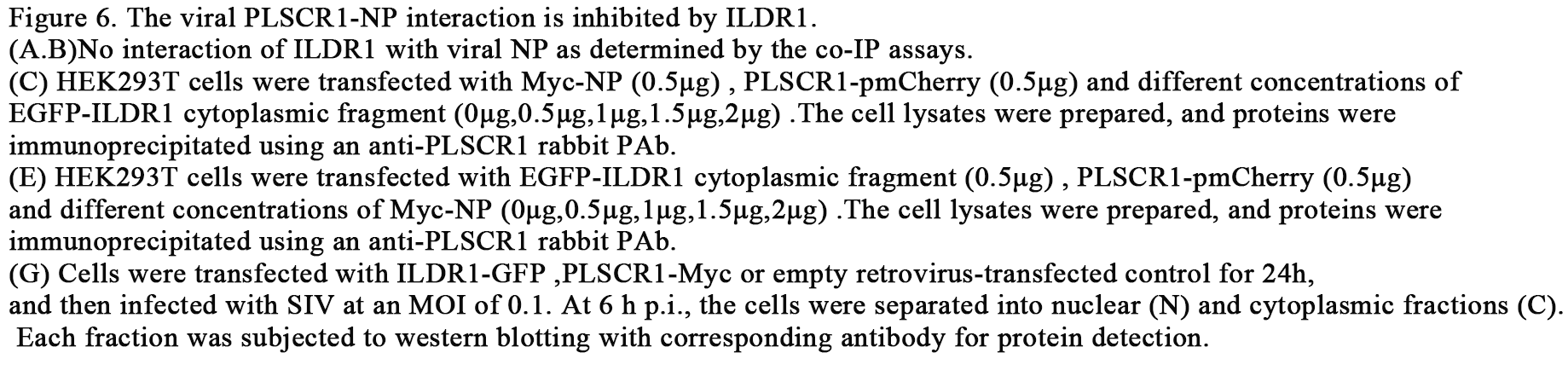

Supplement: Supplementary file 1 — Supplementary Information. [file 41598_2022_12598_MOESM1_ESM.docx]
